# Emphysème segmentaire géant congénital compressif: diagnostic et traitement

**DOI:** 10.11604/pamj.2016.23.173.8529

**Published:** 2016-04-13

**Authors:** Moussa Abdoulaye Ouattara, Seydou Togo, Bourama Kané, Sadio Yena

**Affiliations:** 1 Service de Chirurgie Thoracique Hôpital du Mali, Bamako, Mali; 2 Service de Pédiatrie Hôpital du Mali, Bamako, Mali

**Keywords:** Emphysème segmentaire, congénital, segmentectomie, Segmental emphysema, congenital, segmentectomy

## Abstract

L'emphysème lobaire géant congénital est une pathologie malformative rare du nourrisson. Les auteurs rapportent un cas similaire qui se distingue par son siège segmentaire encore plus rare et son caractère compressif chez qui une segmentectomie a été réalisée en urgence avec succès pour lever la détresse respiratoire dans un pays en développement.

## Abstract (en)

Giant congenital lobar emphysema is a rare pathological malformation in infants. Authors report a similar case that is remarkable for its even rarer segmental location and compressive character. The patient underwent successful urgent segmentectomy for the treatment of respiratory distress in a developing country.

## Introduction

Les malformations broncho-pulmonaires (MBP) résultent d'accidents de développement du système broncho-pulmonaire. Ce sont des affections rares et polymorphes parmi lesquels l'emphysème lobaire géant congénital (ELG) représente 3 à 15% [1, 2]. L'emphysème segmentaire géant congénital est une variante topographique encore plus rare de l'ELG, pouvant également évoluer vers la détresse respiratoire. Le cas que nous rapportons est un emphysème segmentaire géant congénital diagnostiqué tardivement et dont le siège segmentaire a été découverte peropératoire. Le traitement réalisé a été une décompression en urgence par exérèse du culmen dans un pays en voie de développement.

## Patient et observation

**Figure 1:**
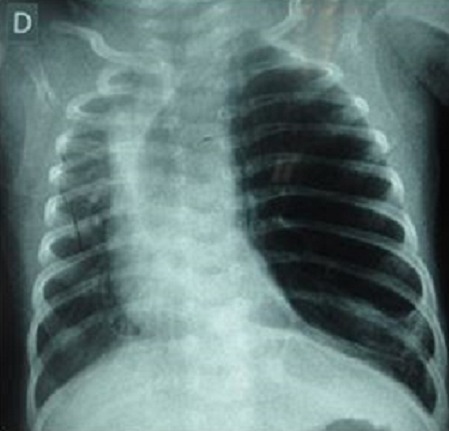
Radiographie du thorax préopératoire

Il s'agit d'un enfant de 4 mois de sexe masculin, 3^ème^ enfant de la fratrie, issu d'une grossesse à terme au cours de laquelle des échographies réalisées n'ont pas retrouvé de malformation congénitale. Il a été référé du service de pédiatrie pour pneumothorax compressif. Dans ces antécédents il existe une notion de dyspnée à la naissance sans notion de souffrance fœtale ayant évolué favorablement sous oxygénothérapie. En revanche des petites crises de dyspnée associées à la toux qui survenaient de façon intermittentes depuis la naissance ont été traitées médicalement. Ailleurs l'interrogatoire n'a pas retrouvé de notion de malformation dans la famille. A son admission le patient présentait une détresse respiratoire avec une fréquence respiratoire à 28 cycles par minute, une saturation en oxygène en air ambiant à 82%, une tachycardie à 130 pulsations par minute, un bon état général avec un poids à 6 kg pour une taille à 58 cm. L'examen physique a révélé une asymétrie thoracique avec un tirage intercostal, sus et sous sternal. Un tympanisme gauche a été retrouvé à la percussion associée à une diminution homolatérale des murmures vésiculaires. La radiographie thoracique de face réalisée dans le service de pédiatrie montrait une hyperclarté homogène avec une distension l'hémithorax gauche, une diminution de la trame vasculaire pulmonaire et une déviation du médiastin vers le coté controlatéral (Figure 1).

**Figure 2:**
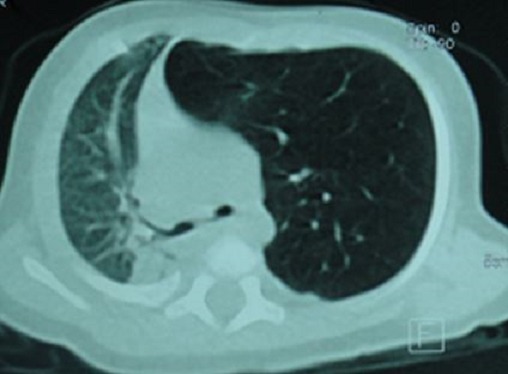
Scanner thoracique préopératoire

**Figure 3:**
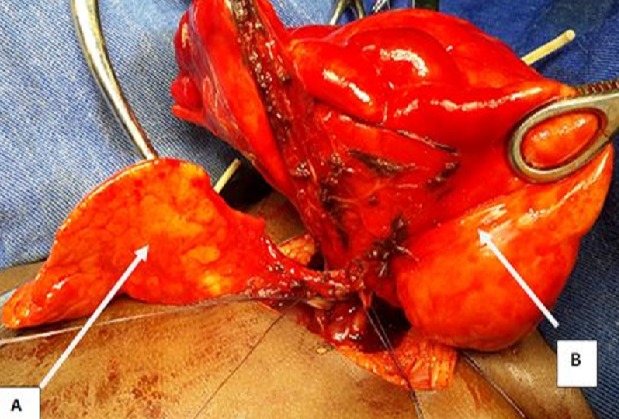
Image peropératoire: (A) lingula; (B) culmen

**Figure 4:**
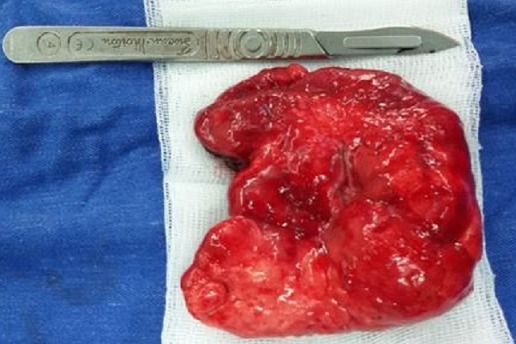
Pièce opératoire

**Figure 5:**
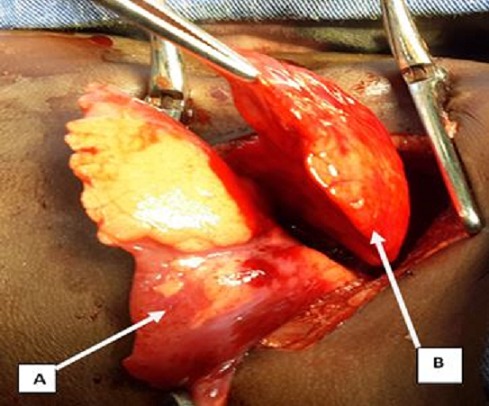
Réexpansion pulmonaire: (A) lobe inférieur; (B)lingual

**Figure 6:**
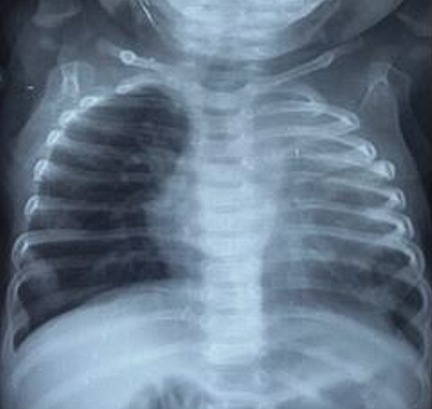
Radiographie du thorax postopératoire

La tomodensitométrie thoracique a permis de préciser sa topographie lobaire supérieure gauche (Figure 2). L'endoscopie bronchique et la scintigraphie pulmonaire n'ont pas été réalisées. Une indication de lobectomie supérieure gauche a été proposée et la préparation a consisté à une oxygénothérapie, des séances de nébulisations au salbutamol et à la réalisation d'un bilan biologique préopératoire. Au bloc opératoire nous avons réalisé sous anesthésie générale avec une intubation trachéale classique, une thoracotomie antérolatérale. En peropératoire nous avons découvert un emphysème segmentaire géant au dépend du culmen (Figure 3). La Culminectomie a été réalisée de façon classique avec de petites périodes d'apnées pour faciliter la dissection (Figure 4). Une réexpansion du lobe inférieur et de la lingula a été observée (Figure 5). L'examen anatomopathologique postopératoire de la pièce n'a pas montré de bouchons muqueux, de corps étrangers mais une bronche segmentaire culminale de calibre réduit. Au plan microscopique le parenchyme pulmonaire présentait des dilatations alvéolaires sans modification de sa structure. Dans les suites opératoires, il a présenté une pneumopathie apicale gauche ayant évolué favorablement sous traitement médical. La sortie a été effectuée à J7 postopératoire après la réalisation d'une radiographie thoracique de contrôle (Figure 6). Actuellement à 6 mois de cette intervention il s'agit d'un nourrisson ayant une bonne croissance avec des paramètres respiratoires satisfaisants.

## Discussion

L'ESG est une variante topographique rare de l'ELG. Le diagnostic anténatal ELG est rarement fait [1, 2]. Cette malformation se révèle habituellement dans les premiers jours ou mois de vie [1–3]. En effet, les manifestations cliniques apparaissent à la naissance dans 33% des cas et avant l’âge d'un mois dans 50% des cas [2, 3]. Dans notre cas, il y a eu une errance diagnostique, le patient a été pris en charge pour une pneumopathie communautaire récidivante sans examen radiologique, ce qui explique la découverte tardive malgré la précocité des signes cliniques. La découverte tardive est également en rapport avec l'insuffisance de ressources humaines spécialisées. La dyspnée est le signe clinique le plus fréquent [3]. Elle est souvent d'installation progressive, évoluant dans un contexte d'apyrexie faisant évoquer une origine malformative. En cas de retard diagnostic elle peut évoluer vers une détresse respiratoire par compression des structures adjacentes, pouvant mettre en jeu le pronostic vital [3]. Le diagnostic a été suspecté par la radiographie du thorax et confirmée par la tomodensitométrie thoracique. Cependant le siège culminal a été un diagnostic peropératoire, ceci s'explique par l'importance de la distension qui a rendu difficile l'identification de la segmentation anatomique du poumon homolatéral.

La scintigraphie pulmonaire bien qu'elle ne soit pas disponible dans notre pratique, est un examen important qui permet de visualiser des troubles de la ventilation et de la perfusion au niveau du lobe emphysémateux [2]. Quant à la bronchoscopie, elle a un intérêt étiologique et thérapeutique mais peut être grave risquant d'augmenter l'hyperdistension. Elle permet d’éliminer la présence d'un corps étranger intra-bronchique, un bouchon muqueux ou de rechercher une anomalie bronchique pouvant être responsable de l'emphysème. Dans le cas présent la détresse respiratoire a constitué une limite à son utilisation. Notre cas s'inscrit dans les 40% des cas où aucune étiologie n'est retrouvée dans la survenue de la surdistension [1, 3, 4]. Quelques rares d'ELG ont été décrits [1–3] mais nous avons trouvé très peu de publication sur cette variante topographique segmentaire qui présente les mêmes risques évolutifs. En raison des retentissements du segment distendu sur le médiastin, le parenchyme pulmonaire homolatéral et controlatéral, la chirurgie semble être le traitement radical parce qu'elle permet de lever la compression [3, 5, 6]. Elle a l'avantage de permettre des exérèses anatomiques segmentaires donc une épargne parenchymateuse plus importante que dans le forme lobaire, d'autant plus qu'il s'agit d'un enfant qui vient à peine au monde.

## Conclusion

L'emphysème segmentaire géant congénital est une forme clinique topographique de l'emphysème lobaire géant ayant les mêmes risques évolutifs et une prise en charge similaire.
